# Dietary supplementation of Neem (*Azadirachta indica*) leaf extracts improved growth performance and reduced production cost in broilers

**DOI:** 10.14202/vetworld.2020.1050-1055

**Published:** 2020-06-11

**Authors:** Torun Kumar Paul, Md. Mehedi Hasan, Md. Anowarul Haque, Sudipta Talukder, Yousuf Ali Sarker, Mahmudul Hasan Sikder, Mohammad Abu Hadi Noor Ali Khan, Mohammed Nazmus Sakib, Alip Kumar

**Affiliations:** 1 Department of Pharmacology, Faculty of Veterinary Science, Bangladesh Agricultural University, Mymensingh-2202, Bangladesh; 2 Population Medicine and AMR Laboratory, Department of Medicine, Faculty of Veterinary Science, Bangladesh Agricultural University, Mymensingh-2202, Bangladesh; 3 Department of Surgery and Theriogenology, Sher-e-Bangla Agricultural University, Dhaka, Bangladesh; 4 Department of Pathology, Faculty of Veterinary Science, Bangladesh Agricultural University, Mymensingh-2202, Bangladesh; 5 Department of Animal Science, Faculty of Animal Husbandry, Bangladesh Agricultural University, Mymensingh-2202, Bangladesh; 6 Department of Animal Science, School of Environmental and Rural Science, University of New England, Australia

**Keywords:** alternatives to antibiotics, broiler chickens, growth performance, neem leaf extracts, production cost

## Abstract

**Background and Aim:**

Global trend to remove the antibiotic growth promoter (AGP) from animals contributes to the exploration of successful measures to sustain production and reduces the intestinal diseases in the post-AGP era. Plant extracts, therefore, have been used to improve performance and intestinal health. Here, we conducted a study to evaluate the effects of neem (*Azadirachta indica*) leaf extracts (NLE) as alternatives to AGPs in broiler chickens.

**Materials and Methods:**

Sixty day-old broiler chicks were assigned to 12-floor pens, each stocked with five birds and divided into three treatment groups of four pens per treatment. The groups were: Negative control, basal diet without additives; positive control, basal diet with antibiotics and vitamins; and NLE treated group, basal diet supplemented with 0.1% aqua extract of neem leaves.

**Results:**

Overall feed intake was significantly (p≤0.05) highest in the positive control. Higher body weight, higher dressing percentage, and lower feed conversion ratio were observed in birds treated with NLE compared to the negative control group (p≤0.05) but not the positive control group (p>0.05). There was no significant variation in hematology between different groups. Furthermore, the economic evaluation indicated that the NLE treatment was found cheaper than control and antibiotic treatment in cost-benefit analysis.

**Conclusion:**

We suggest NLE might be a cheaper alternative to antibiotics in broiler production as indicated by improved body weight and feed efficiency.

## Introduction

Antibiotics have been added to animal feeds since 1950, to improve feed utilization and growth of farm animals to meet the animal protein demand throughout the world [1]. Consecutively, a wide number of growth promoters have been triggered to use in intensive poultry production systems [2]. The poultry industry has massively adopted the use of antibiotic growth promoters (AGPs) [3] to improve the health of birds by enhancing gut health for better nutrient utilization and feed conversion ratio (FCR) [4]. However, continuous use of AGPs could result in antibiotic residues in meat and develop antibiotic-resistant bacteria [5,6]. Consequently, the use of these AGPs has been discouraged in many countries after banning by the European Union since 2006 [7]. Antibiotic-free alternative substances and strategies for the promotion of livestock growth and disease prevention, particularly in pig and chickens have been investigated over the years [8,9]. In this prospect, herbal products have perceived increased attention in recent years due to their acceptance as natural additives by consumers [10].

Medicinal plants such as *Nigella sativa* L., *Mentha arvensis* L., *Mentha pulegium* L., *Zingiber officinale*, *Allium sativum*, and *Azadirachta indica* (*A. indica*, commonly known as neem) are used from an ancient civilization, to combat diseases in humans, and are being used in poultry rations as natural feed additives for improving growth performance [11-13]. Neem is well known as one of the most versatile medicinal plants, for more than 2000 years and still regarded as “village dispensary” in the Indian ­subcontinent, including Bangladesh, which has a broad spectrum of biological activity [14]. Leaves, seeds, and barks of neem trees have been demonstrated as antimicrobial activity against Gram-positive and Gram-negative bacteria along with immunomodulatory, anti-inflammatory, antifungal, and antioxidant properties [14,15]. Furthermore, the beneficial influences of neem leaves on growth performance [16], carcass characteristics [4], hematological parameters [16,17], and immune responses [18] were also reported in broiler chickens. Moreover, Sarker *et al*. [19] observed a combined effect of neem as a growth promoter and anti-colibacillosis compound in broilers. Pandey *et al*. [15] correlated the phytochemical, antioxidant, and antibacterial attributes of neem leaves through *in vitro* experiment.

Despite these findings, there has been a dearth of information on *in vivo* trials for the growth-promoting effects of neem compared with AGP along with their residual effects on broiler chickens. Therefore, we conducted the study to investigate the efficacy of neem leaf extracts (NLE) as an alternative to AGPs and its cost-effectiveness in broiler chickens.

## Materials and Methods

### Ethical approval

The experimental protocol was approved by the Animal Ethics Committee of the Faculty of Veterinary Science, Bangladesh Agricultural University (BAU) [Approval no.: AWEEC/BAU/2017(12)].

### Study location and period

The study was accomplished at the Laboratory of the Department of Pharmacology (DOP), Faculty of Veterinary Science, BAU, from July to December 2017.

### Processing of neem leaf extracts

Mature fresh neem leaves were harvested from the medicinal plant garden of DOP, BAU, and were identified by the Department of Agronomy, BAU. The infusion of NLE was prepared by the decoction method described by Leila [20] with few modifications. In brief, after collection, leaves were air-dried for 7 days followed by oven-dried at 55°C for 72 h. Afterward, the leaves were finely ground to make fine powder, 5% solution was prepared by dissolving the leaves powder in boiled water and kept overnight covering with aluminum foil paper. Finally, the mixture was filtered using Whatman 40 filter paper (Thomas Scientific), and the solution was administered as an NLE to broiler chicken.

### Experimental design and husbandry

A total of 60 Cobb-500 broiler chicks, irrespective of sex, were collected from Nourish Poultry and Hatchery Ltd., Bangladesh. Standard management conditions were executed for all the treatment groups for floor space, temperature, relative humidity, ventilation, and light. Birds were reared on deep litter where rice husks were used as bedding materials. The chicks were brooded at 35°C on the 1^st^ week, and thereafter the temperature was reduced by 3°C every week until the temperature reached at room temperature (23-25°C). At day 3, birds were weighed and randomly assigned to 12-floor pens with five birds per pen. A randomized complete block design was used with three treatment groups of four pens in each group, and growth-related parameters of all the groups were studied for an overall of 35 days. The groups were: Negative control, basal diet (Table-1) without any additives; positive control, basal diet with antibiotics, Vitamin B-complex, and amino-acid supplementation; and NLE treated group, basal diet supplemented with 0.1% aqua extract of neem leaves (20 ml of 5% NLE per liter drinking water). Data were recorded until day 35. Corn soybean meal based commercial feed (Nourish Feeds Ltd., Bangladesh) was used as basal diet, including a starter (crumble), grower (pellet), and finisher (pellet) at days 3-14, days 15-25, and days 26-35, respectively. Enrofloxacin (days 3-7), and ciprofloxacin (days 8-35) were administered to the birds of positive control following the manufacturer’s recommended dose through drinking water. NLE was administered to the neem treated birds, once daily from days 3 to 35. All the birds were sacrificed at day 35, through incising carotid arteries, and dressing percentage, relative organ weight (percentage of live weight) of liver, heart, pancreas, gizzard, and spleen were recorded.

**Table-1:** Nutrients composition of experimental basal diets.

Composition^1)^	Starter	Grower	Finisher
Moisture % (Max)	12	12	12
Crude protein % (Min)	20	19	18
Fiber % (Max)	5	5	5
Calcium % (Min)	0.95	0.95	0.9
Phosphorus % (Max)	0.45	0.45	0.42
Methionine (Min)	0.45	0.45	0.42
Lysine (Min)	1.05	1.05	1
Metabolizable energy (kcal/kg) (Min)	3000	3050	3100

Max=Maximum, Min=Minimum.

1) Corn soybean meal based commercial diets were used to meet the nutrient requirements of Cobb-500 broilers

### Growth performance and carcass traits

Body weights (measured in the morning before providing feed and water) and mortality rates were recorded daily, and mean body weight was calculated weekly (days 3-35). Feeding was recorded daily, and feed consumption and FCR (feed intake/weight gain) were calculated accordingly. No mortality was recorded during the experimental period. Carcass yield was calculated after slaughtering the birds, by dividing eviscerated weight by live weight. Dressing percentage (dressed weight/live weight) was calculated by weighting after removing the proventriculus, gizzard, small intestine, abdominal fat pad, and heart.

### Hematological assay

Hematological parameters were analyzed at day 35. Fresh blood samples were collected from the wing vein of each bird with EDTA to measure packed cell volume (PCV), hemoglobin (Hb) concentrations, and total erythrocyte count (TEC). PCV was estimated by the microhematocrit method using capillary glass tubes, and Hb concentration was determined by the Hellige Hemometer method. All parameters were determined as the methods described by Lamberg and Rothstein [21].

### Screening of residues by thin-layer chromatography (TLC)

Breast meat samples from all the experimental birds were collected on slaughter and screened for enrofloxacin and ciprofloxacin residue using TLC. TLC plate (MN-Germany), TLC tank, and UV detection box (UV light: F18W-Germany) were used as the TLC apparatus. TLC was performed according to the protocol described previously with required adjustments [22]. TLC plate (20 cm×20 cm) was cut into a suitable size (10 cm×6.66 cm) and a horizontal line of the border was drawn across it from 1.5″ of the bottom edge with a pencil. Another straight line from the 1″ upper edge was also drawn across the plate. Three points were marked in the bottom-line, and 50 μl of each analyte were spotted using capillary glass pipettes. TLC plates were then submerged immediately in the TLC tank contained mobile phase (Acetone and Methanol: 1:1) with the lid covered. After the mobile phase crosses the upper line of the TLC plate, it was dried and was visualized in UV (256 nm) detection box. Then, retention factor (Rf) was calculated by measuring the distance traveled by the solvent, and the distance traveled by individual sample spots. The same Rf value of samples and standards was considered a similar compound.

### Statistical analysis

The data were recorded in a Microsoft Excel and analyzed using SPSS version 20.0 (SPSS Inc, Chicago, Illinois, U.S.A). The negative control, positive control, and treated groups of chickens were compared by the statistical analysis of variance, in a completely randomized design. When a significant effect of the treatment was detected, Fisher’s least significant difference test was used to make pairwise comparisons between means and considered statistically significant at p≤0.05.

## Results

### Growth performance

The effects of the dietary treatments on body weight, daily feed intake, and FCR are shown in Table-2. During the rearing period, birds fed with NLE supplement had significantly (p≤0.05) higher mean body weight at days 21, 28, and 35 compared to the negative control. There was no significant (p>0.05) difference observed in body weight between the birds receiving NLE and antibiotic treatment groups. Feed intake was significantly higher in the negative control group compared to other groups at days 3-14, and the positive control group had higher feed intake compared to other groups at days 3-35 (p≤0.05). Birds treated with NLE had improved FCR at all ages compared to the negative control (p≤0.05). Moreover, FCR was not different in the NLE treatment group when compared with the positive control group except from days 3 to 7.

**Table-2:** Effects of dietary treatments on the performance of broilers at different ages.

Growth parameters	Age (days)	Dietary supplements		

		NC	PC	NLE treatment
Body weight (g)	3-7	93.5	89.5	100.5
	3-14	305	303	322
	3-21	639^b^	685^a^	703^a^
	3-28	1127^b^	1238^a^	1256^a^
	3-35, overall	1547^b^	1782^a^	1833^a^
Feed intake (g/day)	3-7	110	110	111
	3-14	496^a^	460^b^	454^b^
	3-21	1041	1041	991
	3-28	1770	1770	1733
	3-35, overall	2384^b^	2478^a^	2378^b^
FCR	3-7	1.18^a^	1.23^a^	1.10^b^
	3-14	1.63^a^	1.52^a,b^	1.41^b^
	3-21	1.63^a^	1.52^a,b^	1.41^b^
	3-28	1.57^a^	1.43^b^	1.38^b^
	3-35, overall	1.54^a^	1.39^b^	1.29^b^

NC=Negative control, PC=Positive control, NLE=Neem leaf extracts, FCR=Feed conversion ratio.

a,b Values in the same row not sharing a common superscript differ significantly (p≤0.05)

### Carcass traits

Dressing percentage, relative organ weights, and absolute organ weights are shown in Table-3. The mean dressing percentage values of NLE treated birds were significantly (p≤0.05) higher than the negative control; however, it had no significant difference compared to the positive control. In terms of organ weights, significantly (p≤0.05) highest mean of heart weight, spleen weight, pancreas weight, relative heart weight, and relative liver weight was observed in the NLE treated group. Moreover, NLE-treated group had significantly (p≤0.05) higher relative pancreas weight and lower relative gizzard weight compared to the positive control.

**Table-3:** Effects of experimental diets on dressing percentage, organ weights, and their relative weights of broiler chickens.

Parameters (g)	NC	PC	NLE treatment
Dressing percentage	60.15^a^	64.22^b^	63.56^b^
Liver weight	45.20	51.29	51.67
Relative liver weight	1.63^a^	1.65^a^	1.82^b^
Gizzard weight	18.62	20.00	21.47
Relative gizzard weight	0.64^a^	0.78^b^	0.73^a^
Heart weight	10.26^a^	9.84^a^	12.32^b^
Relative heart weight	0.41^a^	0.42^a^	0.55^b^
Spleen weight	2.28^a^	2.40^a^	3.07^b^
Relative spleen weight	0.09	0.09	0.11
Pancreas weight	3.44^a^	3.06^a^	3.85^b^
Relative pancreas weight	0.13^a,b^	0.12^a^	0.15^b^

NC=Negative control, PC=Positive control, NLE=Neem leaf extracts.

a,b Means with different letters in the same row differ significantly (p≤0.05)

### Hematological parameter of broiler chickens

The hematological analysis of broilers is summarized in Table-4. There were no significant (p>0.05) variation in hematological (TEC, Hb, and PCV) parameters of broiler chickens at day 35 between the groups.

**Table-4:** Comparison of total erythrocyte count, hemoglobin, and packed cell volume in different groups.

Dietary treatments	Blood parameters		

	TEC (×10^6^/μl)	Hb (g/dl)	PCV (%)
Negative control	2.48	7.00	21.00
Positive control	2.93	7.50	24.75
NLE treatment	2.87	7.60	26.33

TEC=Total erythrocyte count, Hb=Hemoglobin, PCV=Packed cell volume

### Economic evaluation

The economic evaluation depicted that maximum profit per kg live weight broiler was in NLE-treated group (BDT 35.58 ≈ $ 0.42) compared to the positive control (BDT 30.60 ≈ $ 0.36) and negative control (BDT 19.26 ≈ $ 0.23) (Table-5).

**Table-5:** Effects of different treatments on the economics of broilers at the end of 5 weeks trial.

Description	NC	PC	NLE treatment
Cost/chick (BDT)	39.00	39.00	39.00
Average feed consumed/bird (kg)	2.384	2.478	2.378
Average live weight/bird (kg)	1.55	1.78	1.83
Cost of feed/kg (BDT)	44.00	44.00	44.00
Cost of *Azadirachta indica* leaves/bird (BDT)	0.00	0.00	1.50
Medicinal (antibiotic) cost/bird (BDT)	0.00	10.00	0.00
Feed cost/bird (BDT)	104.90	109.03	104.63
Miscellaneous cost/bird (BDT)	20.00	20.00	20.00
Total cost/broiler (BDT)	163.90	168.03	163.63
Total cost/kg broiler	105.74	94.40	89.42
Sale price/kg live weight (BDT)	125.00	125.00	125.00
Sale price/broiler (BDT)	193.75	222.5	228.75
Net profit/broiler (BDT)	29.85 (0.35*)	54.47 (0.64*)	65.12 (0.77*)
Profit/Kg live weight (BDT)	19.26 (0.23*)	30.60 (0.36*)	35.58 (0.42*)

NC=Negative control, PC=Positive control, NLE=Neem leaf extracts, BDT=Bangladeshi currency.

* US Dollar

### Screening of residue

Antibiotics residue was not detected in chicken meat from the control group and neem treated birds. Ciprofloxacin residue was detected in 55% of birds of the positive control group, but enrofloxacin residue was not found in the positive control.

## Discussion

The growth-promoting effect of NLE is supported by different studies [16,19,23]. The body weight gain in NLE fed chickens could be possibly due to the diversified effect of NLE on intestinal microflora, and the presence of macro and micro minerals such as potassium, magnesium, phosphorous, iron, copper, manganese, and zinc in *A. indica* leaves [24,25]. Similar findings were also reported by Tipu *et al*. [26], who found better weight gain using *A. indica* fruit as a feed additive. However, Nayaka *et al*. [17] reported depression in body weight on supplementing neem leaf powder. This may be attributed to the inhibition of feed in birds due to the bitter taste and unpalatability of the neem leaf powder. Growth effects of neem and antibiotic treatment without significant difference in the present study could be explained by the findings of Ansari *et al*. [24], who reported that the growth properties of NLE depend on the concentration and dose-response relationship.

The observation of FCR in our study is also consistent with the previous data [23]. Neem leaves have individual bioactive compounds that can be attributed to the antimicrobial and antiprotozoal properties which can limit the growth and colonization of pathogenic and nonpathogenic bacterial species in chickens gastrointestinal tract [27,28]. Thus, poultry gut could lead to greater digestion efficiency and feed utilization that resulted in improved FCR [4,29]. Perhaps, due to the same reason, significantly highest feed intake was recorded in antibiotic-treated birds. However, a non-significant difference in FCR between the control group and different neem treatments is also reported [17].

The dressing percentage was upgraded in birds fed on neem and antibiotics, which is in line with the findings of Ansari *et al*. [24], who found better dressing percentages in birds fed with herbal plant diets than control. In contrast, Elangovan *et al*. [30] found no effects of neem extracts on carcass characteristics. Moreover, significant variations in internal organs weights were observed with the spleen that had significantly the highest weight in the neem treated group. Recruitment of B and T lymphocytes could happen in NLE potentiated birds that lead to increase lymphoid organ weight as supported by other studies [10,24]. However, non-significant differences in organ weights among the control and treatment groups fed on antibiotics or plant extracts were also reported [4,16]. The variation in different studies may be due to the concentrations of antibiotics and neem or other herbal extracts supplied to the experimental birds. Moreover, the length of the experimental period and slaughtering age also may contribute toward dressing percentage.

A good physiological status of birds can be evaluated based on hematological parameters and their values are varied due to variation of dietary supplements [31]. Various studies found that NLE feeding can significantly change the bird’s hematology [17,24]. However, we have not observed any significant difference in hematology between control and treated groups. This non-significant effect of NLE suggests that NLE does not affect blood cell formation, constituents, and their function. These no effect observation was also reported in TEC, Hb, and PCV values in broiler chickens [16,32]. However, Sarker *et al*. [19] found significantly increased TEC in broilers treated with 1% neem leaves at day 42, probably, due to the different NLE concentration and study period.

Our cost-benefit analysis of the study is in agreement with Ansari *et al*. [2], who also reported extra profit per bird by supplementing *A. indica* plant as a growth promoter in broilers.

No AGP residue was detected in NLE-treated group confirms no contamination or cross-contamination during the experimental period. However, ciprofloxacin residue was detected in positive control birds due to the administration of the antibiotic throughout the experimental period with no withdrawal period. Ciprofloxacin is a commonly used poultry antibiotic and a high frequency of ciprofloxacin residues in chicken meat in Bangladesh was also reported previously [22]. Neglecting, the drug withdrawal period can be an important underlying reason to remain antibiotic residues in meat [33]. This is a major public health concern as most of the farmers in Bangladesh do not follow the withdrawal period of antibiotics during marketing their birds [34]. Enrofloxacin could be transformed into ciprofloxacin by demethylation after administration into the body and maybe, therefore, enrofloxacin residues were not observed in tested samples [35,36]. Moreover, enrofloxacin was only used from days 3 to 7, and its residues may be excreted later from the body that also can support the findings.

## Conclusion

AGP may cause poultry meat residual effects and lead to the development of resistant strains. Irrational use of AGP leads to multidrug-resistant bacteria, a threat for both humans and animals. Here, we report NLE could be a better alternative to AGP in broilers as manifested by highest body weight with the least FCR. *A. indica* leaves extracts, therefore, could be used as an inexpensive growth promoter in broilers. Purification of active ingredients of *A. indica* leaves responsible for antimicrobial effects should be investigated further to identify the active ingredients with pharmacological properties.

### Authors’ Contributions

TKP, YAS, and MHS: Designed the study. TKP, MMH, MAH, ST, and YAS: Conducted the experiment and performed the laboratory works. MMH and ST: Drafted the manuscript. TKP, MMH, and AK: Analyzed and interpreted the data. MAH and MNS: Helped in the acquisition of data and writing the manuscript. MNS: Helped in technical writing. MHS and MAHNAK: Supervised the research. MHS, MAHNAK, and AK: Critically revised the manuscript. All the authors read and approved the final manuscript.
